# A survey of palliative care domains and the palliative care provision confidence of Thai family practitioners

**DOI:** 10.1186/s12904-023-01272-8

**Published:** 2023-10-05

**Authors:** Itthipon Wongprom, Arthit Chaithanasarn

**Affiliations:** Department of Family Medicine, Faculty of Medicine Ramathibodi Hospital, Mahidol University, Rama VI Street, Ratchtevi District, Bangkok, 10400 Thailand

**Keywords:** Palliative care, Family physicians, Care domains, Confidence levels, Curriculum development

## Abstract

**Introduction:**

Despite continuously growing, palliative care in Thailand still needs further development. Many family physicians provide various levels of palliative care. However, there is no information regarding what aspects of palliative care family practitioners provide and how much confidence they have. This study aims to identify gaps between expected care domains and provided care and to reveal the domains in which family physicians are less confident.

**Method:**

This study is a cross-sectional survey study. An online questionnaire was publicly published to recruit target participants, Thai family physicians who have received formal residency training and actively practice medicine. The estimated number of actively practising family physicians was 540 persons. Participants were asked which palliative care domains they provide and to rate their confidence in each. A narrative analysis was performed using two relevant frameworks.

**Result:**

One hundred and seven responses were received. Forty-six per cent of participants have no further training in palliative care other than during their residency programs. At least 63.6 per cent of participants provide palliative care two half-day per week or less. Grief and spiritual care enjoyed the least percentage of the participant’s responses regarding provided care. These two domains also received the lowest percentage of confidence in providing care from the participants.

**Discussion:**

Though they are considered essential domains, there are gaps between expected care and actual care in grief management and spiritual care. Grief and bereavement care are mentioned in the residency curriculum; nonetheless, there is an inconsistency regarding spiritual care. Increasing access to continuous education and integrating it into curriculums should be considered to reduce the gaps. Additionally, a national guideline regarding levels of palliative care and the roles of family physicians will benefit from establishing comprehensive palliative care programmes.

**Supplementary Information:**

The online version contains supplementary material available at 10.1186/s12904-023-01272-8.

## Introduction

Palliative care has grown continuously around the world over the last decade. It is also better standardised widely in both local and international academia. Many countries have invested in establishing high-quality palliative care. For example, the U.K. stresses the importance of the funding model as well as training and education for healthcare providers [1]. In North America, Canadian palliative care associations launched a palliative care model to guide healthcare professionals in developing high-quality palliative care [2]. In Thailand, a new post-graduate palliative care training program has been recently established along with the integration of palliative medicine into the family medicine residency curriculum [3–5].

Despite continuously growing, palliative care in Thailand still needs further development and integration. From 2011 to 2017, the palliative care system evolved from a stage of isolated palliative care provision to a stage of preliminary integration into mainstream service provision, 3b to a4, as categorised by The Worldwide Hospice Palliative Care Alliance [6, 7]. Many hospitals in Thailand provide some parts of palliative care, such as home visits or pain management, while some have more integrated, comprehensive programs. Nonetheless, despite being incorporated into national policy, many health jurisdictions struggle to establish comprehensive palliative care programmes, either in terms of coverage or quality. Consequently, heterogeneity of palliative care provision among health jurisdictions is observed; resources and capacity, both from the perspective of human resources and funding, play an important role [8].

Therefore, understandably, many family physicians provide their patients with various levels of palliative care. They serve alongside general practitioners and other generalists such as internists. It is also important to note the context of medical education in Thailand regarding general practitioners and family physicians. The undergraduate medical degree in Thailand is a 6-year curriculum. Medical graduates will earn a medical degree and independent license to provide medical care as general practitioners. Their practice varies. Some provide family practice, while others offer semi-urgent, acute, and chronic disease care.

Meanwhile, upon obtaining a medical degree and license, general practitioners can have further family Medicine residency training to become family physicians. The residency programs take three years in total, and palliative care is a competency in the curriculum [9]. In 2022 there were 1,341 family physicians in Thailand, considering a doctor-population ratio of 1:50,000. This number was derived from the number of family physicians from various training tracks, including formal training, certifying, and community-based training tracks [10]. A policy shift in 2017 demanded that family physicians participate in the Primary Care Cluster Model to provide more comprehensive care to patients in their catchment areas; however, there were still many challenges, such as doctor retention problems, inadequate resources, and training systems [5, 11]. In 2017, Thailand was categorised as “Palliative care at a preliminary stage of integration”, level 4a [12]. The country is still facing many challenges, such as accessibility, a small proportion of palliative care providers, confidence in communicating with patients and families among some providers, and drug availability [8, 13, 14]. Family physicians play an important role in palliative care accessibility and opioid availability from primary care to tertiary care settings; hence, family physicians’ inadequacy and quality affect palliative care systems as a whole.

It is crucial to tailor the curriculum to better serve health service systems. Hence, input for revising palliative care training or need assessments should be called for. However, there is no information regarding what aspects of palliative care family practitioners provide, how much confidence they have, and the proportion of family physicians providing palliative care. This survey aims to determine the proportion of Thai family physicians who provide palliative care, identify a discrepancy between delivered services and expected competencies, and identify challenging aspects of palliative care among Thai family physicians.

## Method

### Target population and sampling technique

This study is a cross-sectional survey study using an online questionnaire. The target population is Thai family physicians who have received formal residency training and actively practice medicine. Identifying all actively practising doctors is challenging due to a longstanding lack of relevant data. Many physicians no longer provide family practice to patients and families; they provide semi-urgent or speciality care instead. This also applies to many board-certified family physicians. Thus, focusing on a more homogenous group of practitioners, presumably the majority of family practitioners, is appropriate.

Recruitment was conducted by the purposive sampling method. The estimated number of former family medicine residents was 773 persons (“RCFPT: The number of graduated family medicine residents”, 2022). Given a lack of specific information about family physicians, we applied a 30 per cent reduction to reflect more accurate numbers of physicians who actively practise. We adopt this number from a general practitioner’s retention rate as there is a contextual similarity between the two fields of practice [15, 16]. The expected response rate is 50 per cent, accounting for approximately 270 physicians.

### Data collection

Data being collected in this study comprises three parts; two are general information, and one is the confidence in providing care. General information includes prior speciality training, additional training in palliative care, time spent on providing palliative care service, workplace setting, provided palliative care domains, and frequencies of palliative care service that participants provided in each setting: outpatient, in-patient consultation, home care, and palliative care unit. The data collected in the third part are the confidence in providing palliative care in the following domains: disease management, physical symptoms, psychological care, social care, spirituality, grief, and end-of-life care. Participants rated their confidence as 5 levels: Not at all, Not very, Neutral, Somewhat, and Very. The questionnaire was designed by the principal investigator (IW), then was checked for face validity by the co-investigator (AC).

The survey was conducted via a Google form, and the online data is stored in a secured cloud Google Drive provided by Mahidol University. An invitation link was sent to a community of practice messaging groups such as Line application or WhatsApp and online social media forums, including Facebook groups and pages. No participants’ identifiable data is collected. A reminder link will be sent to the same platform four weeks after the first link to increase response rates.

### Data analysis and reporting

A descriptive analysis was applied in this study. Categorical data was presented in frequency and percentage. No participants’ identifiable data were reported.

## Result

### Participants

**Fig. 1:**
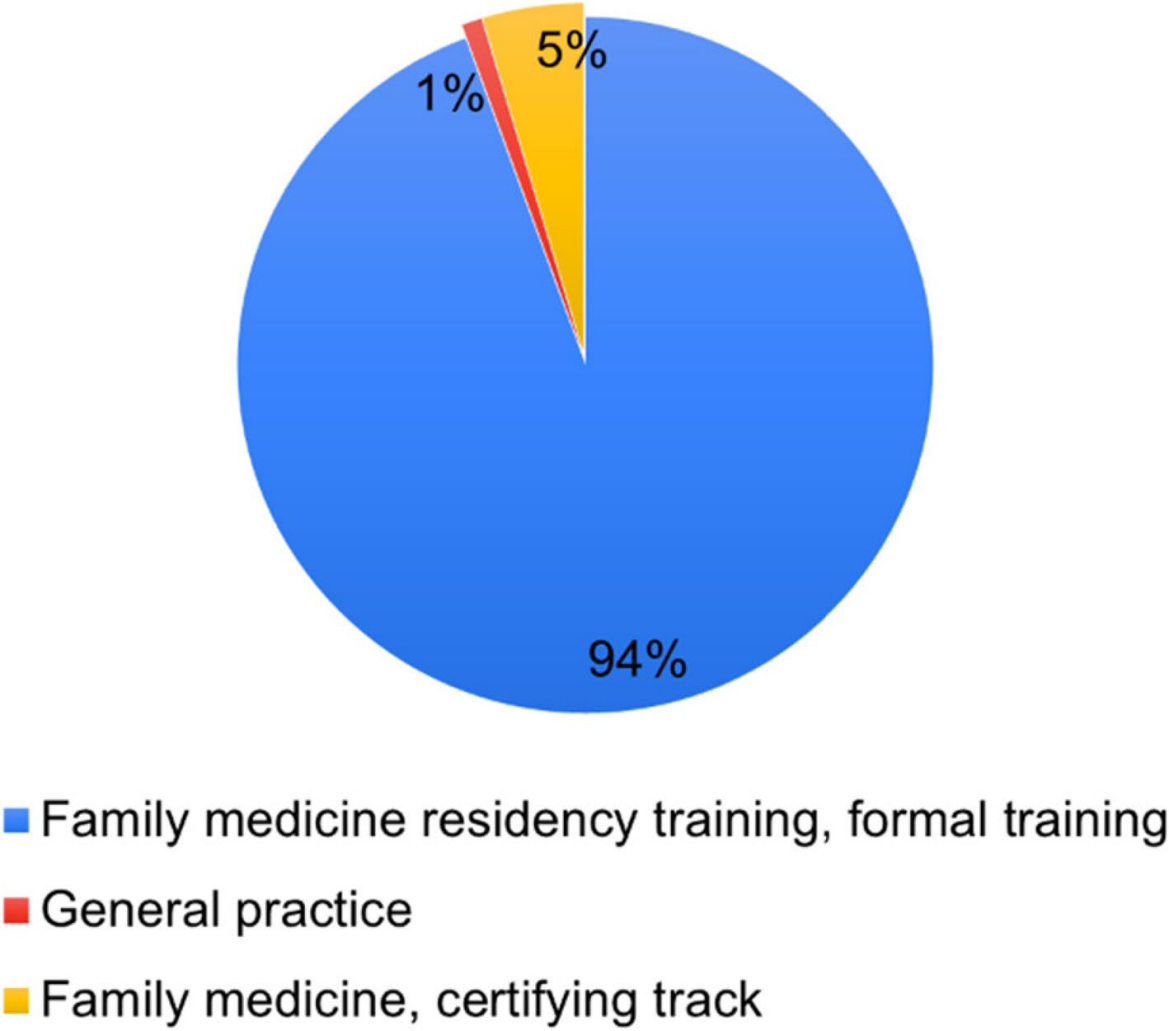
Participants by speciality training

**Fig. 2:**
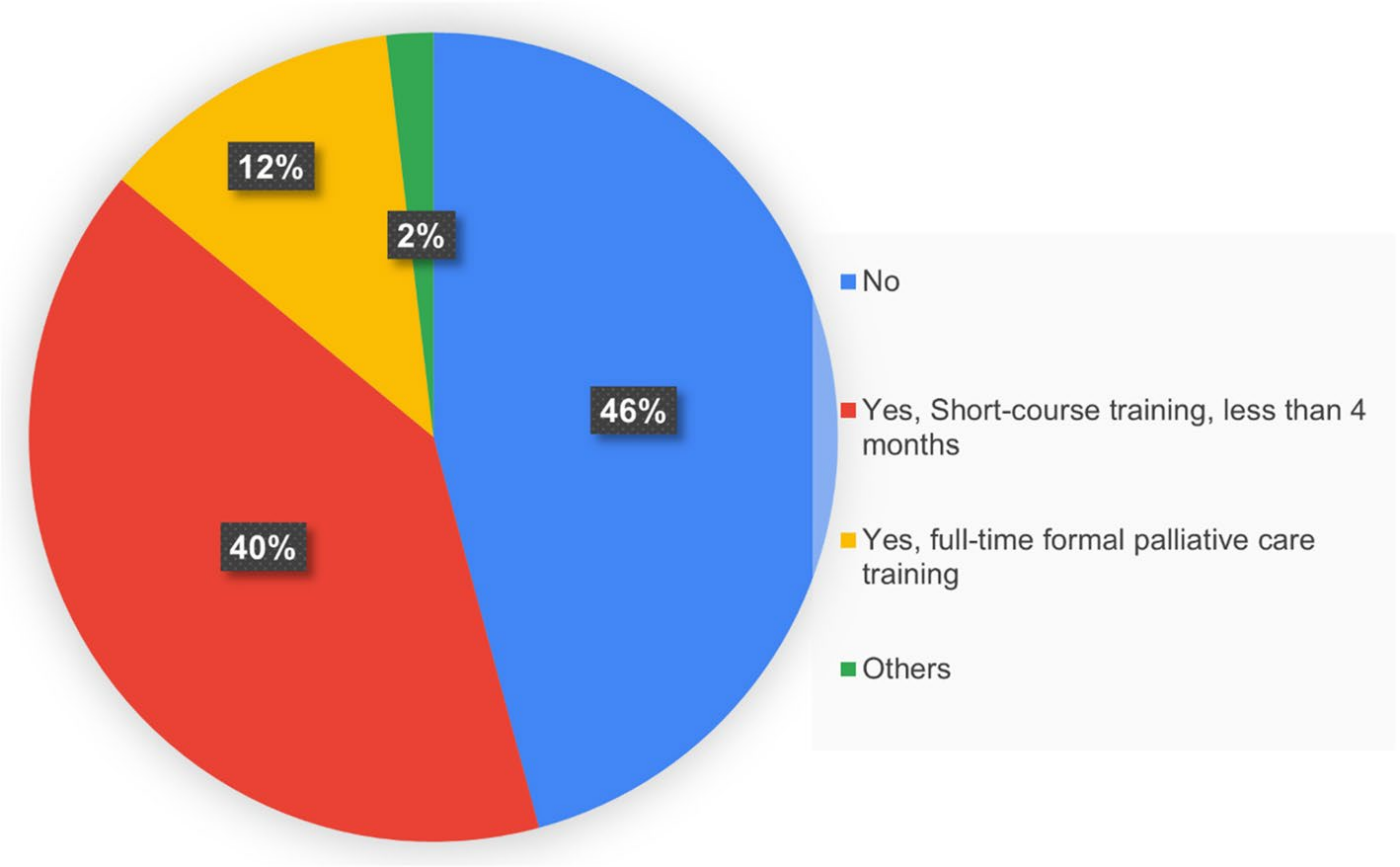
Participants by additional palliative care training

The total number of responses was 107, as in Figs. 1 and 2. There were 100 participants (94.3%) who were family physicians who finished the formal residency training track, while 5 persons (4.7%) were family physicians licensed via the family medicine certifying track. One person (0.9%) is a general practitioner, a medical graduate. Regarding palliative care training, 46.2% of participants have no further training other than during their residency programs. Additionally, 39.6% of participants had short course training shorter than four months, and 12.3% underwent full-time formal training. The rest, 1.8%, have taken other forms of palliative care educational activities.

In workplace settings, roughly two-thirds of participants engage in part-time palliative care, which equates to two half-days per week or less. The distribution of respondents’ workplace locations shows that nearly half are employed in general or provincial hospitals, while 32.1% and 23.6% of participants work in community hospitals and medical school-affiliated hospitals, respectively. There were 5 participants (5.5%) who worked in other settings, such as private hospitals and companies.

### Provided services and confidence levels

**Fig. 3:**
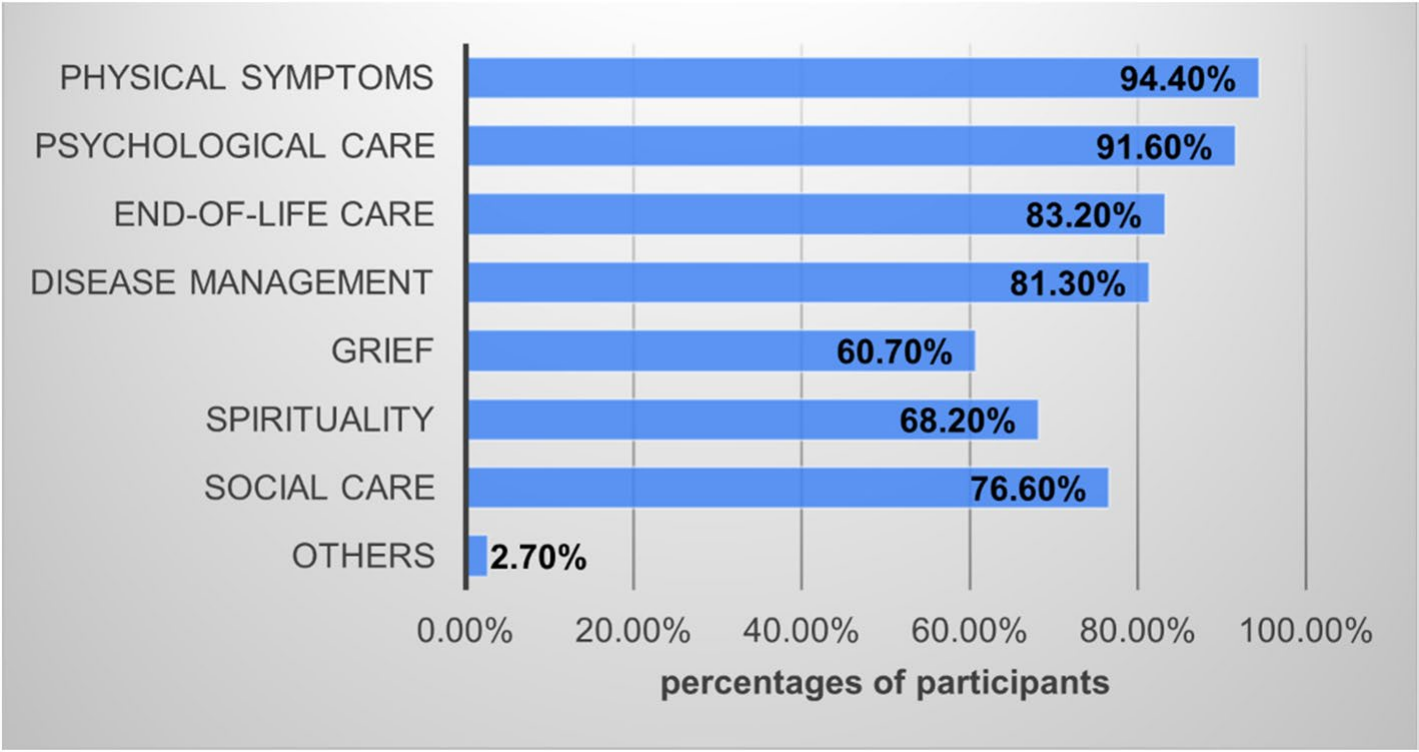
Provided palliative care domains

Participants were asked if they provided services in the following categories: disease management, physical symptoms, psychological care, social care, spirituality, grief, end-of-life care, and other practical issues. The percentages of participants providing each aforementioned palliative domain are shown in Fig. 3. The most common provided care domains are physical and psychological symptoms and end-of-life care. Regarding confidence levels, Fig. 4.1 shows percentages of each level in each care domain, while the combined percentage of “Somewhat” and “Very” was shown in Fig. 4.2, along with other confidence levels. Physical symptoms and end-of-life care enjoyed the most confidence, while grief and spirituality had the lowest percentage.

**Fig. 4:**
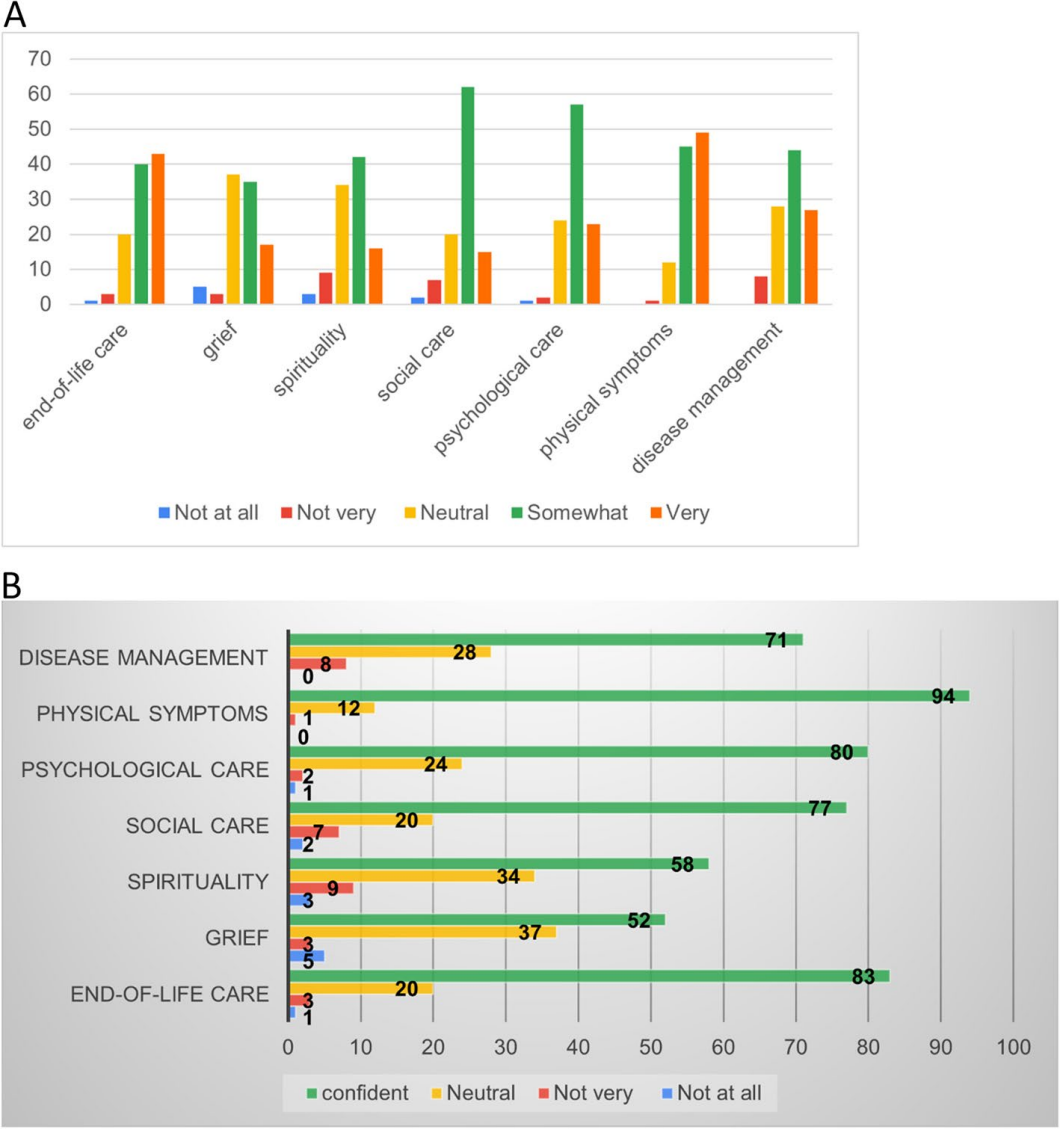
1 Overall confidence levels in each domain. 2 Confidence levels in each domain

## Discussion

As mentioned, symptom management and end-of-life care are the most delivered care domains and enjoyed the most confidence among respondents. Meanwhile, grief and spiritual care are provided less often and enjoy the least confidence. Two frameworks were used to analyse these results. The first framework is the Model to Guide Hospice Palliative Care, Canadian Hospice Palliative Care Association. This model is based on the National Principles and Norms of Practice, which aims to standardise palliative care and facilitate the establishment of hospice palliative care programs. Its contents cover definitions, principles, significance, the norm of practice, and management [2]. The relevant frameworks reside in the Domains of Issues Associated with Illness and Bereavement section. This section provides an overview of essential issues that palliative care providers should focus on, including disease management, physical symptoms, psychological care, social care, spirituality, grief, end-of-life care, and other practical matters. Additional details are provided for each domain. For example, regarding physical symptoms domains, common issues include pain and other symptoms, function, nutrition, wound, and habit. These domains reflect scopes of practice or a standardised care package that patients and families should receive. Furthermore, the narrative analysis focused on both domains that are often provided and those that are overlooked. These domains are similar to other palliative care standard frameworks that have been used widely.

The second framework is the family medicine residency curriculum. As a formal palliative care training program was established in Thailand in 2018, a significant proportion of palliative care is delivered by family physicians [3, 17]. Palliative care has been integrated into many training programs, from undergraduate medical degree curricula to other specialities and family medicine residency training. According to the Royal College of Family Physicians of Thailand’s current curriculum, palliative care competency is a subcategory under the Patient Care Competency. There are seven domains in which learners need to excel after finishing the residency program: palliative care principles, communication skills, common symptoms management, comprehensiveness, bereavement care, interprofessional skills, and appropriate referral [18]. Additionally, palliative care-related Entrustable Professional Activities have been integrated into the curriculum, ensuring all graduates earn adequate proficiency [18, 19]. Toward the third year, the last year of training, residents should be able to practice the following with supervision on demand: symptoms management, end-of-life care, spiritual Health, family meetings and counselling, and doctor-patient-family relationships.

### Expectation and reality: the gaps between provided services and standards

More than 85 per cent of participants reported providing care in the following domains: disease management, physical symptoms, and psychological care. These domains are in accordance with the roles of family physicians in palliative care, according to medical doctor competency and family physician competency levels stated by the Medical Council of Thailand. In particular, pain and symptom management is a fundamental and essential element when delivering high-quality palliative care. According to the Royal College of Family Physicians of Thailand, final-year family medicine residents need to secure an Entrustable Professional Activity (EPA) regarding palliative care at a level that they can practice in most contents with supervision on demand. One of the five skill sets in this EPA is to manage pain, symptoms management and supportive care. Hence, it is understandable that nearly all participants reported their care provisions in physical symptom domains. Additionally, a very high percentage of responses reported psychological care provision. Similarly, this could be explained by family physicians’ nature, identities and designated competencies, especially in Thailand, where they are well-known among other professions for their excellent skills for psychosocial issues.

On the other hand, despite being stated in the EPA as one of the five essential skills, providing grief and bereavement care and spiritual care enjoyed the least percentage of the participant’s responses. Grief and bereavement care clearly illustrate a considerable gap between actual and expected care provision. Though some patients and families may receive such care from other healthcare providers, such as psychiatrists and psychologists, the fact that many family physicians providing palliative care do not provide bereavement care is still worrying. Systematic screening and follow-up with families at risk or with abnormal grief can be done effectively by gatekeepers such as primary care providers and family physicians [20–22]. Regarding spirituality, the percentage itself is another gap that should be addressed.

Nonetheless, it is interesting to witness this high percentage, reflecting that some family physicians provide spiritual care in their practices. Given the lack of spiritual care professions in Thailand, in a caveat of confusing religious approaches with spiritual care in the modern sense, it is imperative to explore further the contents or elements of spiritual care delivered. Indeed, grief and bereavement care and spirituality domains are the prominent gaps between the standards of care and the actual care provided.

### Confidence, competency and curriculums

Regarding confidence in each domain of palliative care, there are two issues worth mentioning. Firstly, grief and spiritual care receive the lowest percentage of confidence in providing care. This concurs with the gaps between provided services and standards mentioned earlier, though this study does not aim to and cannot identify its causation or correlation. Nonetheless, some possible explanations can be raised. With inadequate services, participants may have limited experience providing care, leading to a lack of confidence. By contrast, a lack of confidence in those domains may lead to hesitancy to establish those services. Secondly, physical symptoms and end-of-life care domains are the only two domains that the respondents rated Very confident as the highest percentage. Again, a concordant direction between provided services and confidence is observed. Similar to the assumptive explanation, it is reasonable for family physicians to initially establish a service that they are competent and confident in, symptom management in palliative care [23].

According to the Canadian Hospice Palliative Care Association and the Royal College of Family Physicians of Thailand, similar to many standard frameworks, grief and spiritual care are considered essential domains of palliative care. However, while the EPA proposed by the Royal College of Family Physicians of Thailand explicitly addresses spiritual care and grief management, only grief management is clearly stated in the family medicine training curriculum. In fact, the curriculum, which is the larger umbrella under which EPA falls, should clearly state the aspect of spirituality. The ambiguity may lead to confusing directions of the training and learning activities [24, 25].

### Is curriculum development enough?

It is understandable to start tackling these confidence issues by revising or developing the curriculum. The result from this survey ensures us that the current curriculum is, at the very least, staying in the right direction when providing physical symptoms and psychological care. It also suggests the need to pay more attention to spirituality and grief domains and be consistent throughout the curriculum. Nonetheless, revising the curriculum alone does not seem to be adequate. Several other strategies should be done concomitantly, such as continuing education. British Columbia Centre for Palliative Care proposed a framework for palliative care education and training that consists of four essential pillars [26]. One of them involves increasing accessibility to continuing education. This could be a strategy to provide more confidence in grief and spiritual care. Most continuing education in palliative care in Thailand has focused on symptom management and primary palliative care. Grief and spirituality are not common topics seen in academic conferences.

Another pillar that could relate to confidence issues is a supportive learning environment. Ongoing coaching and mentoring system should be developed to help learners after they finish training programs. Community of practice is an example model in which palliative care physicians or experienced family physicians could contribute their knowledge to novices [27–29]. The Royal College of Family Physicians of Thailand has been offering annual short-course training. Though its primary goal is for palliative medicine fellows, it welcomes all levels of learners and could be developed further to be both an educational resource and a community of practice.

### Levels of palliative care

Though the aforementioned educational approaches are crucial, the bigger question is, who should provide that care? There are several guidelines and frameworks that mention levels of palliative care. Pallium Canada suggests that the intensity of palliative care ranges from a palliative approach to care to specialised palliative care. In Australia, the intensity of care involvement of palliative care specialists versus other providers has been used to identify the complexity and level of care needs [30]. Unfortunately, however, there has not been an established guideline regarding the care complexity levels and responsible providers in Thailand.

**Fig. 5:**
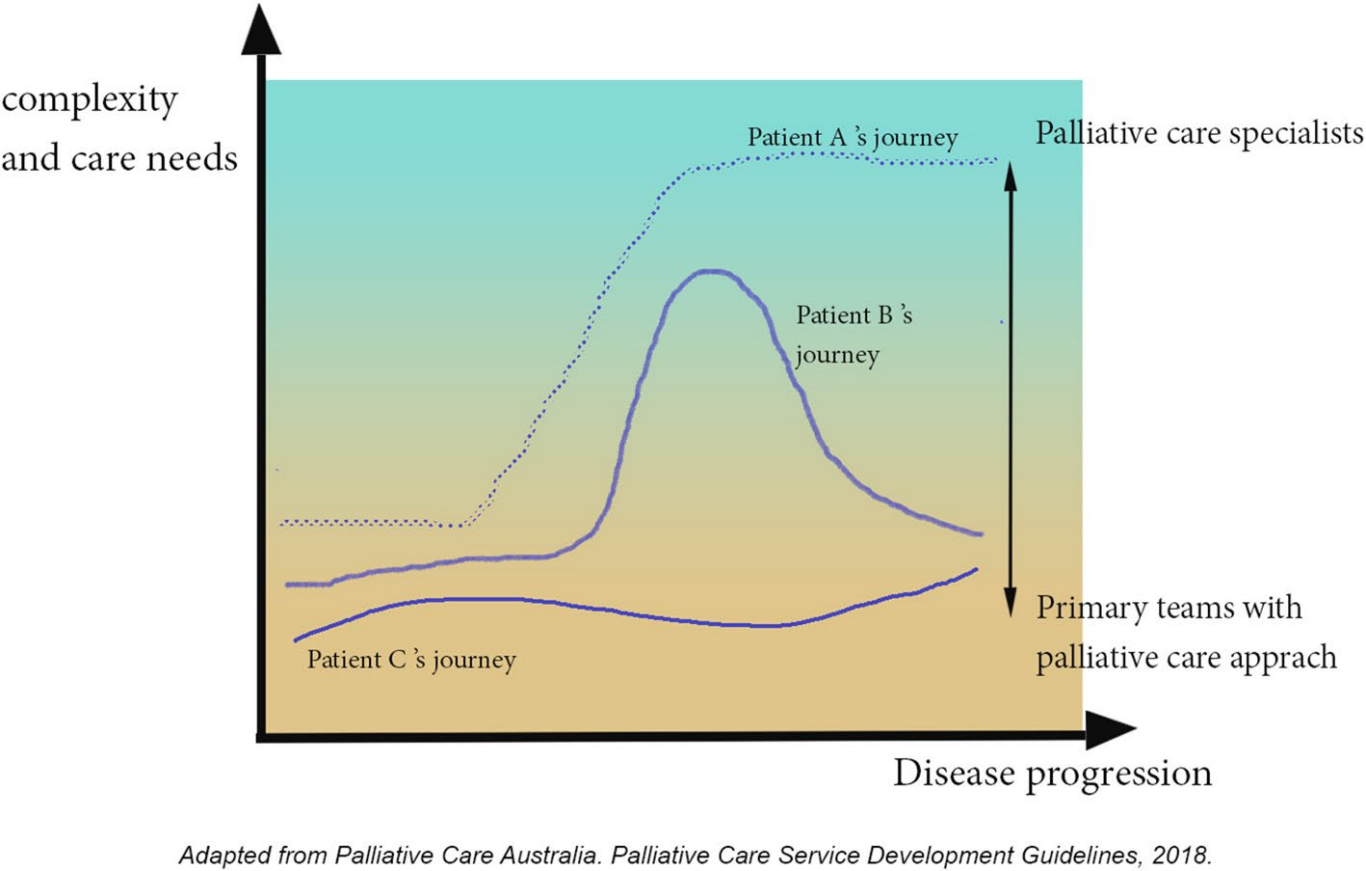
Levels of palliative care and care teams

It is understandable to rely on generalists and family physicians to manage symptoms due to the inadequacy of palliative care physicians in Thailand. Nonetheless, it is questionable that grief and spirituality issues should be emphasised more in the curriculums and continuing education. British Columbia’s palliative care education framework also proposes competency-based education guidelines in its first pillar. There is no formal guideline in Thailand from governmental organisations such as the Ministry of Public Health or professional institutes such as the Royal College of Family Physicians. It would be beneficial and more apparent if the stakeholders established a competency-based guideline before considering curriculum revision. An example of the guideline is illustrated in Fig. 5.

### Limitation

Two issues are considered a limitation in this research. The first issue is that the response rate is below our expectations. This could be a concern when analysing the data as it might not represent the target population well. Nonetheless, it should be noted that there is inadequate information regarding the number of actively practising family physicians. In Thailand, general medical practice is provided by general practitioners, family physicians, and other generalists, such as internists and nurse practitioners. Family physician community and family medicine are still in a developing phase [31]. Some change their career to others, while others focus on administration or managerial levels. Hence, it is challenging to estimate the number accurately. The second issue is the methodology of this research. As it is a cross-sectional survey, it is not designed to demonstrate correlations or causations of the results.

### Future research

Since this study provided information about the gaps of knowledge and proposed directions to close them, it is imperative to further explore each palliative care domain. For example, understanding which parts of grief care are challenging for family physicians could be a helpful need assessment before launching new educational programs. To elaborate, some might feel that it is difficult to screen and communicate about pathological grief, while others may have trouble with making an effective follow-up system. Additionally, future studies could focus on how other specialists perceive the abovementioned level of palliative care and competency at each level and their needs to develop appropriate educational programs.

## Conclusion

Palliative care is a rapidly growing field worldwide, including in Thailand. This research contributes information regarding types of services, confidence in providing care, and palliative care domains. The result identifies the gaps between the expected and actual care provided and the confidence in providing care in each domain. Symptom management and end-of-life care are the most common services provided by family physicians. They also enjoy the majority of confidence level. Meanwhile, bereavement care and spiritual care are the domains that are least provided and with the least confidence level. The narrative analysis in this research suggests that the gaps should be approached by revising the training curriculum, developing continuing education, and writing a clear policy about the levels of palliative care.

### Supplementary Information

**Additional file 1: Appendix A.** The survey questionnaire. **Appendix B.** A palliative care domain framework. **Appendix C.** The palliative care competency framework.

## Data Availability

The datasets used and analysed during the current study are available from the corresponding author upon reasonable request.

## Abbreviations

EPA: Entrustable Professional Activity
RCFPT: The Royal Colledge of Family Physicians of Thailand
