# Supplementary material for: A survey of palliative care domains and the palliative care provision confidence of Thai family practitioners
Source: BMC Palliat Care. 2023 Oct 5;22:147. doi: 10.1186/s12904-023-01272-8 (PMC10552241; doi:10.1186/s12904-023-01272-8)
Supplement: Supplementary file 1 — Additional file 1: Appendix A. The survey questionnaire. Appendix B. A palliative care domain framework. Appendix C. The palliative care competency framework. [file 12904_2023_1272_MOESM1_ESM.docx]

**Appendix A**

The survey questionnaire

**Information about this survey**

This is a cross-sectional survey using an online questionnaire. We aim to identify discrepancies between delivered services and expected competencies among Thai family physicians. Also, we would like to identify challenging aspects of palliative care provision. This could provide insight into palliative care service coverage provided by family practitioners and be used as a need assessment for formal curriculum designs and continuous professional development.

The questionnaire takes 5-10 minutes. Your identifiable personal data will not be asked in this survey. Participants will answer the survey anonymously. Data will be stored only on a secured University of Toronto OneDrive student account. We invite all family physicians who have completed family medicine residency training to take part in this survey. Please note that this participation is entirely on voluntary basis. There will be no consequences whether you decide to take the survey or otherwise.

I agree to participate in this survey.

**Part 1: General information**

Which of the following best describes your training program?

Family medicine residency training, formal training
  Family medicine, certifying track

other speciality residency training

Do you have additional training in palliative care?
 no
  yes, full-time formal palliative care training
  yes, Short-course training, less than four months

others, please specify

How much time do you provide palliative care?
 Full-time  more than 2 half days a week  2 half days a week or less

What is your work setting?

Community hospital  Provincial hospital  Medical school’s hospital

Private hospital  others, please specify _______

**Part 2: provided services**

|  | **N/A** | **1-2 half days/ week** | **2-5 half days/ week** | **> 5 half days/ week** |
| --- | --- | --- | --- | --- |
| **Outpatient** |  |  |  |  |
| **In-patient consultation** |  |  |  |  |
| **Home care** |  |  |  |  |
| **Palliative care unit** |  |  |  |  |

**Which domains do you provide? (check all that apply)**

**disease management** **physical symptoms**  **psychological care**  **social care**

**spirituality**  **grief**  **end-of-life care**  **others, please specify _______**

**Part 3: confidence in providing care**

| **Item** | **Not at all** | **Not very** | **Neutral** | **Somewhat** | **Very** | **NA** |
| --- | --- | --- | --- | --- | --- | --- |
| **disease management** |  |  |  |  |  |  |
| **physical symptoms** |  |  |  |  |  |  |
| **psychological care** |  |  |  |  |  |  |
| **social care** |  |  |  |  |  |  |
| **spirituality** |  |  |  |  |  |  |
| **grief** |  |  |  |  |  |  |
| **end-of-life care** |  |  |  |  |  |  |
| **Other, ____________** |  |  |  |  |  |  |

**Appendix B**

**A palliative care domain framework**

**DISEASE**

**MANAGEMENT**

Primary diagnosis, prognosis,

Secondary diagnoses

(e.g., dementia,

psychiatric diagnoses, substance

use, trauma)

Co-morbidities (e.g.,

delirium,

seizures, organ failure)

Adverse events (e.g.,

side effects,

toxicity)

Allergies

*
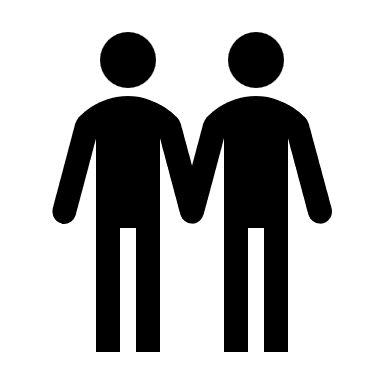
*

Patients and families facing with life-threatening illness

**END OF LIFECARE/**

**DEATH MANAGEMENT**

Life closure (e.g., completing

business, closing relationships, saying

goodbye)

Gift giving (e.g., things, money,

organs, thoughts)

Legacy creation

Preparation for expected death

Anticipation and management of

physiological changes in the last

hours of life

Pronouncement, certification

Perideath care of family,

Funerals

Rites, rituals

**SOCIAL**

Cultural values, beliefs, practices

Family caregiver protection

Guardianship, custody issues

Routines, rituals, recreation, vocation

Financial resources

Legal

Relationships and roles

Isolation, abandonment

Privacy, intimacy

Safe, comforting environment

Activities of daily living

Dependentstransportation

**PSYCHOLOGICAL**

Personality, strengths, behaviour,

motivation

Depression, anxiety

Emotions (e.g., anger, distress,

hopelessness, loneliness)

Fears (e.g., abandonment, burden,

death)

Control, dignity, independence

Con_ict, guilt, stress, coping

responsesdelirium,

seizures, organ failure)

Adverse events (e.g.,

side effects,

toxicity)

Allergies

**SPIRITUAL**

Meaning, value

Existential, transcendental

Values, beliefs, practices, af_liations

Spiritual advisors, rites, rituals

Symbols, iconsdelirium,

seizures, organ failure)

Adverse events (e.g.,

side effects,

toxicity)

Allergies

**PHYSICAL**

Pain and other symptoms*

Function, safety, aids:

Motor (e.g.,mobility, swallowing,excretion)

Senses (e.g., hearing, sight, smell,

taste,touch)

Physiologic (e.g., breathing,

circulation)

Sexual, Wounds

**LOSS/GRIEF**

Loss

Grief (e.g., acute, chronic, anticipatory)

Bereavement planning

Mourning

*Adapted from A Model to Guide Hospice Palliative Care, Canadian Hospice Palliative Care Association* (2)*.*

**Appendix C**

**The palliative care competency framework**

| **EPA Title** | Palliative care | | | | | | |
| --- | --- | --- | --- | --- | --- | --- | --- |
| **Description of the activity** | Providing care for palliative patients and families, either at clinics, in-patient, or home setting | | | | | | |
| **Expected KSA** | 3.1 Pain, symptoms management and supportive care  3.2 End-of-life care (advance directive care, living will, grief and bereavement)  3.3 Spiritual Health  3.4 Family Meeting and counselling  3.5 Doctor-patient-family relationship | | | | | | |
| **Link with competencies**  **and predefined**  **milestones)** | EPA | PGY 1 | | PGY 2 | PGY 3 | | |
|  | level | Not allowed to  practice the EPA | Practice in some content with  supervision on  demand | | | Practice in most contents with  supervision on  demand |  |
| **Sources of information**  **to determine progress** | - Home care log  - palliative care case report  - Palliative care case conference | | | | | | |
| **Basis for formal**  **entrustment decision)** | - palliative care case report  - Written exam (MCQ, MEQ) and OSCE  -workplace-based assessment (MINI-CEX, 360 degree  assessment) | | | | | | |

*Adapted and translated from The royal college of family physicians of Thailand's family medicine residency training curriculum* (4)
